# Eryptosis Indices as a Novel Predictive Parameter for Biocompatibility of Fe_3_O_4_ Magnetic Nanoparticles on Erythrocytes

**DOI:** 10.1038/srep16209

**Published:** 2015-11-05

**Authors:** Qian Ran, Yang Xiang, Yao Liu, Lixin Xiang, Fengjie Li, Xiaojun Deng, Yanni Xiao, Li Chen, Lili Chen, Zhongjun Li

**Affiliations:** 1 Department of Blood Transfusion, The Second Affiliated Hospital, The Third Military Medical University, Chongqing, China; 2 Department of Hematology, The Second Affiliated Hospital, The Third Military Medical University, Chongqing, China

## Abstract

Fe_3_O_4_ magnetic nanoparticles (Fe_3_O_4_-MNPs) have been widely used in clinical diagnosis. Hemocompatibility of the nanoparticles is usually evaluated by hemolysis. However, hemolysis assessment does not measure the dysfunctional erythrocytes with pathological changes on the unbroken cellular membrane. The aim of this study is to evaluate the use of suicidal death of erythrocytes (i.e. eryptosis indices) as a novel predictive and prognostic parameter, and to determine the impact of Fe_3_O_4_-MNPs on cellular membrane structure and the rheology properties of blood in circulation. Our results showed that phosphatidylserine externalization assessment was significantly more sensitive than classical hemolysis testing in evaluating hemocompatibility. Although no remarkable changes of histopathology, hematology and serum biochemistry indices were observed *in vivo*, Fe_3_O_4_-MNPs significantly affected hemorheology indices including erythrocyte deformation index, erythrocyte rigidity index, red blood cell aggregation index, and erythrocyte electrophoresis time, which are related to the mechanical properties of the erythrocytes. Oxidative stress induced calcium influx played a critical role in the eryptotic activity of Fe_3_O_4_-MNPs. This study demonstrated that Fe_3_O_4_-MNPs cause eryptosis and changes in flow properties of blood, suggesting that phosphatidylserine externalization can serve as a predictive parameter for hemocompatibility assay.

With rapid progress in nanotechnology, many nano-size materials have been extensively used in biomedical and pharmaceutical industry and industrial production^1^. Nevertheless, the rapid growth of nanotechnology has raised biological safety concerns because of the unique dimensional and physicochemical properties of the nano-size materials^2^. Although the mechanism of toxicity of nanomaterials is complicated and markedly different from that of traditional biomaterials, current evaluations of nanotoxicology are still confined to testing the compatibility of materials using traditional methodologies. A standard research protocol to evaluate the nanotoxicity is lacking, severely limiting the development and applications of nanoparticles^3^. Therefore, there is an urgent need to develop a sensitive, accurate and reliable evaluation system to assess nanotoxicity and to understand its mechanisms.

Fe_3_O_4_ magnetic nanoparticles (Fe_3_O_4_-MNPs) is the only nanomaterial that has been approved for clinical applications because of their relative safety, unique magnetic responsiveness, and their simple and controllable preparation^4^,^5^. Up until now, Fe_3_O_4_-MNPs have been widely explored in the clinic fields, such as magnetic resonance imaging (MRI) contrast agents, biosensors, tumor-targeting photothermal therapy, and regenerative medicine^6^,^7^,^8^,^9^. For example, commercially available Fe_3_O_4_-MNPs (e.g. Feridex) have been used as MRI contrast agent with recommended concentration of 0.56 mg Fe/kg^10^. Although studies concerning the potential risks of Fe_3_O_4_-MNP have been reported^11^, the biocompatibility evaluation relies mainly on *in vitro* cytotoxicity such as hemolysis testing, cell viability, oxidative damage, inflammatory reactions, and genotoxicity, or on pharmacokinetics, and *in vivo* bio-distribution^12^.

Erythrocytes are the main components in the circulation system and are also one of the first components that Fe_3_O_4_-MNPs contact when the nanoparticles are administered through intravenous injection. Fe_3_O_4_-MNPs are generally regarded as hemocompatible based on very low hemolytic activity^13^. Hemolysis testing is a well-accepted classical assay for acute toxicity screening in evaluating hemocompatibility and can reflect the breakage to the erythrocyte membrane. However, hemolysis testing cannot indicate the dysfunction of unbroken erythrocytes with pathological changes on cellular membranes. Increased number of erythrocytes with cellular membrane injuries (not broken to cause hemolysis yet) can cause some physiologic derangement or serious health problems, such as anemia, microcirculation dysfunction, and thrombogenic activation^14^,^15^,^16^,^17^. Moreover, various clinical disorders are associated with excessive injured red blood cells (RBCs) with cellular membrane changes leading to exposure of phosphatidylserine. This process is a contributing factor for several serious diseases, including heart failure-associated anemia^18^, chronic renal failure^19^, hemolytic uremic syndrome^20^, Wilson’s disease^21^, and malignancies^22^. Impairments in erythrocyte deformability have been associated with diabetes mellitus-related renal failure, sepsis, and hypertension^23^. Therefore, it is important to detect the early process of erythrocyte damage before hemolysis.

On the other hand, accumulated evidence in recent years suggests that some nanoparticles have an adverse effect on erythrocytes pre-hemolysis after interacting with RBC *in vitro* and *in vivo*. Owing to the induction of specific structural changes in the lipid bilayer, these nanoparticles caused erythrocyte shape transformation^24^, decreased the deformability and oxygen-delivering ability^25^, modified the heme conformation^26^, and changed hemorheological properties^27^. Although it has been reported that MgNPs-Fe_3_O_4_ (100 μg/ml) can cause cellular membrane damage in cultured lung epithelial cells^28^, the impact of Fe_3_O_4_-MNPs at safe dose on cellular membrane of erythrocytes and circulatory properties beside hemolysis remains unknown.

Eryptosis, also known as programmed erythrocytes death and defined similarly as nucleated cell apoptosis^29^, is characterized by cell shrinkage, phosphatidylserine exposure, membrane blebbing, intracellular Ca^2+^ level increase, elevated ROS (reactive oxygen species) levels, as well as the rapid consumption of ATP. Eryptosis has been used as metric to evaluate the effects of drugs^30^, heavy metal ions^31^, fungi and bacterial toxins^32^,^33^, plant extracts^34^, and pathological factors^35^ on the cellular structure and function of erythrocytes. Thus far, eryptosis has not been investigated as a possible detection index to assess nanoparticle-blood compatibility during clinical application of Fe_3_O_4_-MNP. In nucleated cells, oxidative stress, DNA damage, cell cycle arrest, autophagy, and apoptosis are important parameters for evaluation of nanotoxicity^36^; in RBC, the parameters associated eryptosis also can be used to measure erythrocytes injury that cannot be evaluated by hemolysis analysis.

In this study, we evaluated the use of eryptosis indices as a predictive and prognostic parameter to assess the effect of Fe_3_O_4_-MNPs on RBCs in the cellular function, the cellular membrane microstructure, and flow properties of blood in clinic setting.

## Results

### Fe_3_O_4_-MNPs synthesis and characterization

**Figure 1:**
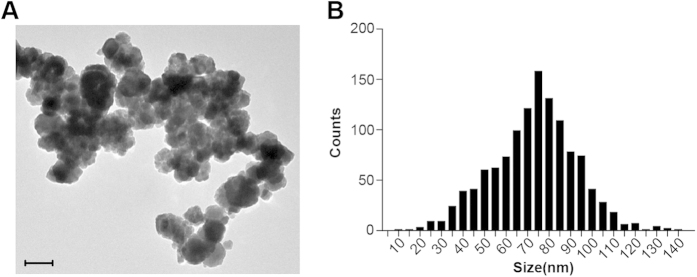
The characterization of Fe_3_O_4_-MNPs. (**A**) An image of Fe_3_O_4_-MNPs morphology obtained by TEM, scale bar = 100 nm. (**B**) The distribution of particle size of Fe_3_O_4_-MNPs. The data were quantified from TEM micrographs.

Fe_3_O_4_-MNPs were prepared by aging ferrous hydroxide gels at elevated temperatures. On the basis of TEM micrographs, Fe_3_O_4_-MNPs displayed a spheroid-like shape with a relatively uniform size. Meanwhile, its size distribution analysis confirmed that the diameter of Fe_3_O_4_-MNPs centered around 72.6 ± 0.57 nm (n = 1200), while 58.17% Fe_3_O_4_-MNPs were between 60 and 90 nm in diameter (Fig. 1A,B). Meanwhile, the average particle size of Fe_3_O_4_-MNPs was measured using DLS. Fe_3_O_4_-MNPs dispersed in water had a hydrodynamic size of 88.78 nm (Figure S 1). Additionally, result from UV-vis absorption spectra suggested that Fe_3_O_4_-MNPs had no characteristic absorption peak (Figure S 2).

### Dose- and time-dependent hemolytic activity of Fe_3_O_4_-MNPs

As shown in Figure S 3A and S 4, after 3 h incubation the Fe_3_O_4_-MNPs at the concentration of 100, 800 and 1,600 μg/ml resulted in 1.78%, 4.08% and 7.81% erythrocyte lysis, respectively. When the incubation time was extended to 24 h, the nanoparticles at the concentration of 100, 200, and 400 μg/ml led 4.44%, 6.81% and 12.34% erythrocyte lysis (Figure S 3A,B and S 4). These results indicated that the hemolytic rate was affected by Fe_3_O_4_-MNPs in a dose- and time-dependent manner. Accordingly, the images of erythrocytes collected after incubation with Fe_3_O_4_-MNPs for 24 hours showed that hemolysis was visible (seen by unaided eye) when Fe_3_O_4_-MNPs reached a concentration greater than 400 μg/ml (Figure S 3C).

Moreover, the blood gas analysis showed that the standard bicarbonate, TCO_2_, and HCO_3_^−^ were increased significantly after incubation with Fe_3_O_4_-MNPs, suggesting a decreased affinity of CO_2_ (Table S1). However, most of parameters, including pH, K^+^, Ca^2+^, Na^+^, Mg^2+^, pO_2_, and pCO_2_, changed slightly after treating with Fe_3_O_4_-MNPs at 200 μg/ml for 48 hours.

### Impact of Fe_3_O_4_-MNPs on RBCs morphology

**Figure 2:**
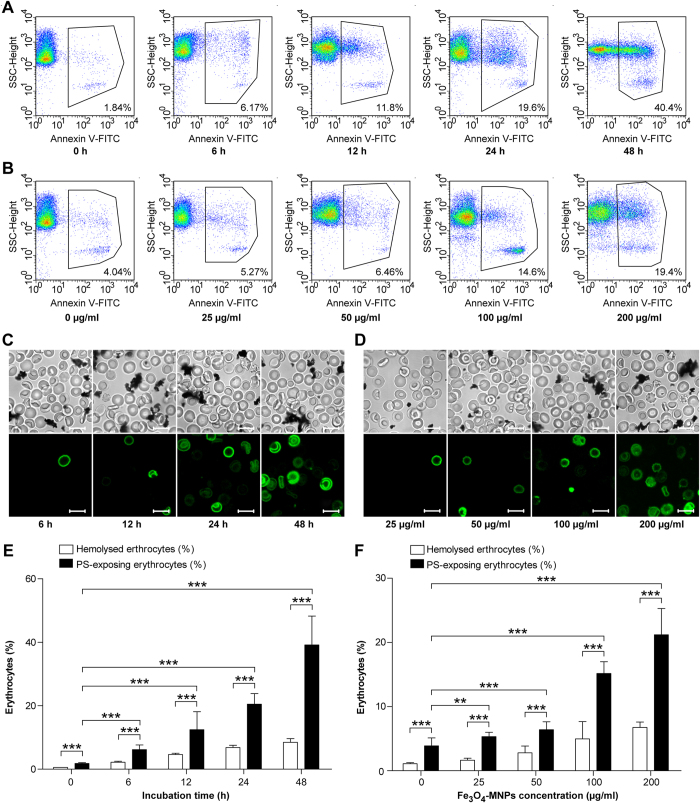
Eryptotic activity of Fe_3_O_4_-MNPs. Percentage of eryptosis for cells incubated with Fe_3_O_4_-MNPs at (**A**) different time points (0, 6, 12, 24, and 48 h) and (**B**) different concentrations (0, 25, 50, 100, and 200 mg) determined by flow cytometry. Annexin V – phosphatidylserine confocal images of erythrocytes incubated with Fe_3_O_4_-MNPs at (**C**) different concentrations and (**D**) time points. Scale bars = 10 μm. The comparison between hemolytic and erypotic RBCs at different concentrations (**E**) and time points (**F**). Values represent means ± SEM, n = 9, **p < 0.01, ***p < 0.001.

The externalization of phosphatidylserine can be quickly and reliably detected by fluorescent annexin V conjugates^37^, while no study has reported the use of annexin V for evaluation of Fe_3_O_4_-MNPs caused eryptosis. To evaluate Fe_3_O_4_-MNPs induced injury, phosphatidylserine exposure was quantified by flow cytometry and fluorescent imaging. When RBCs were exposed to 200 μg/ml Fe_3_O_4_-MNPs, the number of annexin V-positive erythrocytes increased gradually after 6, 12, 24, and 48 h incubation (Fig. 2A,C). As shown in Fig. 2A, the percentage of PS-displaying cells almost reached 40% at 48 h. And the percentage of PS-displaying cells was increasing along with the increase of Fe_3_O_4_-MNPs concentration (Fig. 2B,D). After exposure with Fe_3_O_4_-MNPs at the concentration of 25 μg/ml for 24 h, 95% of the erythrocytes still had an intact cellular membrane; however, the percentage of phosphatidylserine exposing cells was statistically higher than that of control group.

Additional experiments were performed to elucidate the differences between hemolysis and eryptosis caused by Fe_3_O_4_-MNP exposure. The hemolytic grade was lower than 5% after incubation with the Fe_3_O_4_-MNPs at a dose of 100 μg/ml for 24 hours. The corresponding percentage of PS-displaying erythrocytes was 15.1 ± 1.85%. Consistent with the notion of excellent hemocompatibility, Fe_3_O_4_-MNPs displayed remarkably low hemolytic activity. However, erythrocytes with annexin V positive were three times more than hemolytic erythrocytes (Fig. 2E,F). Annexin V binding is indicative of alterations in the cell surface characteristics, phospholipid organization, and membrane integrity. As a result, the injured cells are recognized, phagocytized, and degraded by macrophages^38^.

### Eryptosis induced by Fe_3_O_4_-MNPs

**Figure 3:**
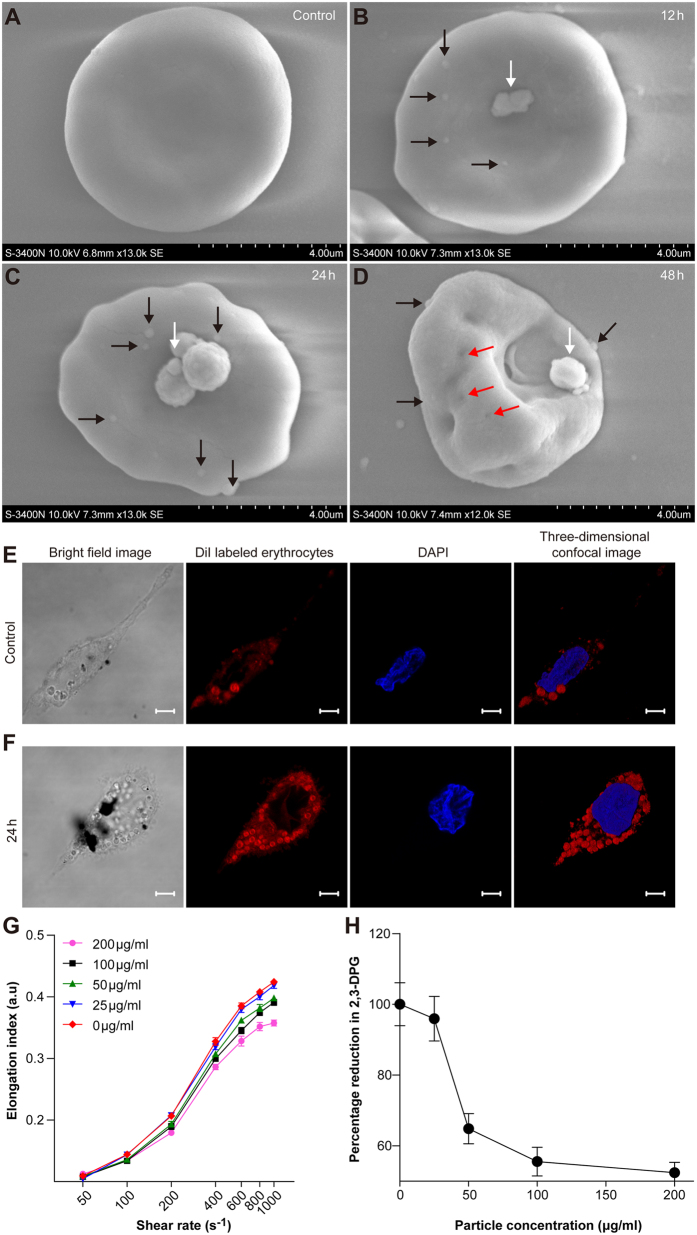
Impact of Fe_3_O_4_-MNPs exposure on erythrocyte membrane, deformability, cytosolic 2,3-DPG and THP-1 phagocytosis of erythrocytes. (**A**–**D**) shows time-dependent impact of Fe_3_O_4_-MNPs on erythrocyte membrane shape and blebbing. White, black, and red arrows indicate Fe_3_O_4_-MNPs, bubbles, and pores, respectively. (**E,F**) shows THP-1 phagocytosis of erythrocytes with or without Fe_3_O_4_-MNPs incubation, respectively. Scale bars = 5 μm. Red: DiI labeled erythrocytes, blue: DAPI labeled nuclear material. RBCs deformability (**G**) and cytosolic 2,3-DPG (H) decrease induced by 24 h exposure of 0, 25, 50, 100, 200 μg/ml Fe_3_O_4_-MNPs. Values represent means ± SEM, n = 9.

In contrast to the control group (Fig. 3A), the Fe_3_O_4_-MNPs exposure group showed that Fe_3_O_4_-MNPs aggregated, agglomerated, and adhered to the cellular membrane of RBCs (Fig. 3B, white arrow), resulting in various types of transformations of cellular membrane along with the incubation time. The blebbing on cellular membrane (Fig. 3B–D, black arrow) was more tangible after 12-hour exposure of Fe_3_O_4_-MNPs, while pores (Fig. 3D, red arrow) were much more apparent after 48-hour exposure. Subsequently, an obvious perturbation and curvature of the cellular membrane occurred after Fe_3_O_4_-MNPs exposure. These morphological characteristics reflected the changes occurring at metabolic levels, indicating that after incubation with Fe_3_O_4_-MNPs erythrocytes were more prone to Phorbol 12-myristate 13-acetate (PMA)-induced THP-1 cells phagocytosis (Fig. 3E,F). With phosphatidylserine externalization and membrane blebbing, RBCs deformability (represented by elongation index) decreased in a dose-dependent manner. When the concentration reached 50 μg/ml, the elongation indices in most shear rates were significantly different from those of untreated group (Fig. 3G). Meanwhile, the concentration of cytosolic 2,3-Disphosphoglycerate, an indicator of RBCs oxygen delivering capacity, also showed a reduction in a dose-dependent manner (Fig. 3H).

**Figure 4:**
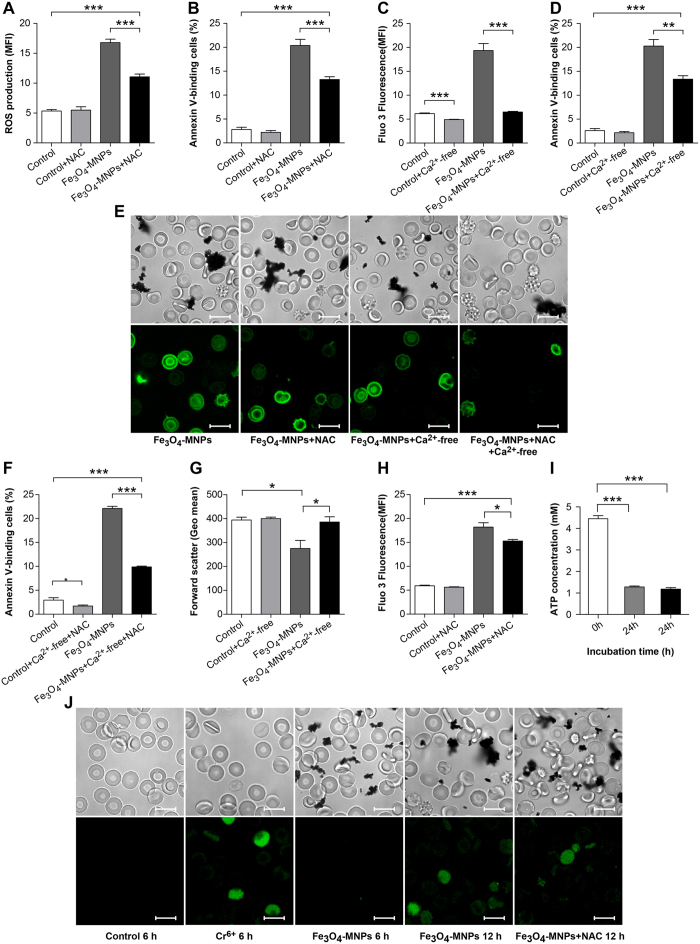
Fe_3_O_4_-MNPs –induced ROS production, Ca2 + influx, ATP consumption, and eryptosis. Intracellular ROS concentrations (**A**) and phosphatidylserine exposure percentages (**B**) induced by 24 h exposure to Fe_3_O_4_-MNPs and protected by NAC. Cytosolic Ca^2+^ concentrations (**C**) and phosphatidylserine exposure percentages (**D**) induced by 24 h exposure to Fe_3_O_4_-MNPs and protected by Ca^2+^-free Ringer solution. (**E**) Annexin V – phosphatidylserine confocal images of erythrocytes incubated with Fe_3_O_4_-MNPs with NAC-, Ca^2+^-free, and NAC- + Ca^2+^-free – Ringer solution. Scale bars = 10 μm. (**F**) Percentage of phosphatidylserine exposure after 24 h exposure to Fe_3_O_4_-MNPs in NAC Ringer solution. Forward scatter (**G**) and cytosolic Ca^2+^ concentration (**H**) of erythrocytes exposed to Fe_3_O_4_-MNPs for 24 h in Ringer solution or NAC Ringer solution. (**I**) Cytosolic ATP concentrations of erythrocytes exposed to Fe_3_O_4_-MNPs for 24 h. (**J**) Confocal images of cytosolic calcium stained by Fluo-3/AM in Ringer solution for 6 h, Cr^6+^ (20 μM) in Ringer solution for 6 h, Fe_3_O_4_-MNPs in Ringer solution for 6 h and 12 h, Fe_3_O_4_-MNPs in N-acetylcysteine added Ringer solution for 12 h. Scale bars = 10 μm, values represent means ± SEM, n = 9, *p < 0.05, **p < 0.01, and***p < *0.001*.

Reactive oxygen species (ROS) is known as an important trigger for eryptosis and ROS antagonist N-acetylcysteine (NAC), an antioxidant agent^39^, can block ROS-mediated eryptosis. Incubation with Fe_3_O_4_-MNPs resulted in significant increases in ROS production and increases in phosphatidylserine exposure. NAC efficiently inhibited Fe_3_O_4_-MNPs-induced ROS production and phosphatidylserine externalization (Fig. 4A,B,E). Ca^2+^ influx is an important signaling mechanism leading to eryptosis. Since Fe_3_O_4_-MNPs exposure also led to high cytosolic Ca^2+^ levels, blocking Ca^2+^ entry by Ca^2+^-free Ringer solution remarkably decreased phosphatidylserine exposure (Fig. 4C–E). It was observed that the inhibitory effect of Ca^2+^-free Ringer solution in combination with NAC was more effective compared to the use of NAC or Ca^2+^-free alone (Fig. 4F).

Mature erythrocytes lack the capacity to store Ca^2+^, and as a result, the Ca^2+^ entry caused by Fe_3_O_4_-MNPs exposure can be readily blocked by Ca^2+^-free Ringer solution. However, Ca^2+^-free Ringer solution could not completely block phosphatidylserine externalization. The percentage of phosphatidylserine exposure in cells in Ca^2+^-free Ringer solution was 13.36% and 20.23% in the presence of Ca^2+^ (Fig. 4B). Furthermore, hexavalent (VI) chromium, an eryptotic trigger causing Ca^2+^ influx, resulted in observable Ca^2+^ entry when exposed for 6 h. However, a detectable increase in cytosolic calcium appeared after Fe_3_O_4_-MNP exposure for more than 12 h, with the cells volume unaltered indicating by forward scatter (Fig. 4G–J). In addition, N-acetylcysteine inhibited ROS production, even with Ca^2+^ entry (Fig. 4H,J). Altogether, these findings indicated that oxidative stress played a pivotal role in Fe_3_O_4_-MNPs induced eryptosis and the initial process of eryptosis involves calcium-independent phosphatidylserine externalization, while Ca^2+^ entry also played an important role in this programmed cell death. In Ringer solution, ATP was consumed rapidly, regardless of whether erythrocytes were treated with Fe_3_O_4_-MNP incubation or not (Fig. 4I) and it was consistent with previous report^40^.

### Blood compatibility of Fe_3_O_4_-MNPs and protective effect of NAC *in vivo*

**Figure 5:**
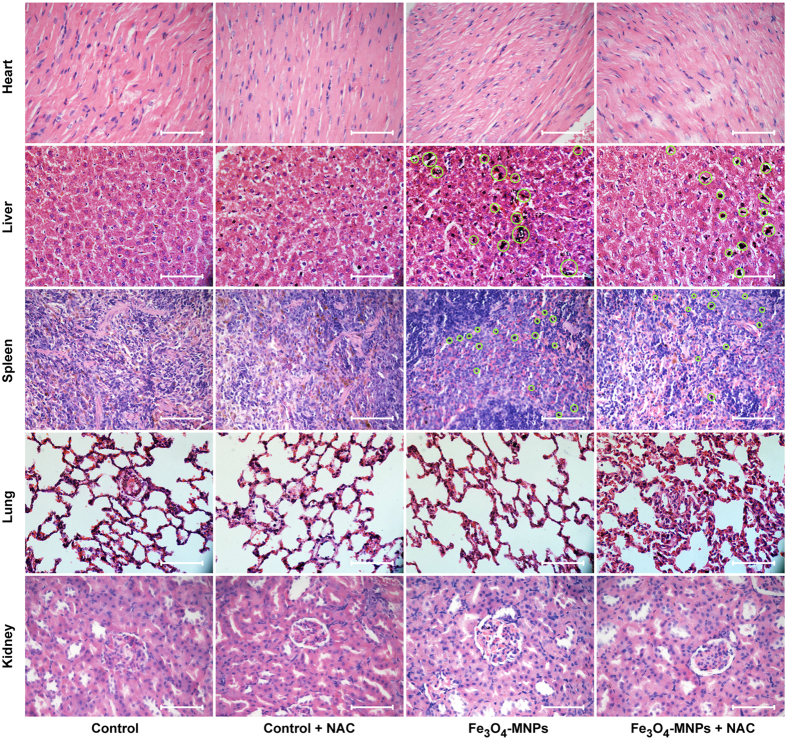
H&E staining on heart, liver, spleen, lung, and kidney samples from rats injected with saline, saline with NAC, Fe_3_O_4_-MNPs, and Fe_3_O_4_-MNPs with NAC. Rats were sacrificed 24 h after the last injection. Aggregated Fe_3_O_4_-MNPs were found in Fe_3_O_4_-MNPs injected groups and indicated by green circles. Scale bars = 50 μm.

To investigate pharmacokinetic characteristics of Fe_3_O_4_-MNPs, blood Fe ions concentration after Fe_3_O_4_-MNPs injection was measured using inductively coupled plasma optical emission spectrometry (ICP-OES). We found that Fe ion concentrations were higher than that in *in vitro* experiment until 6 h (Figure S 5). As compared with the control group, the Fe_3_O_4_-MNPs treated rats showed no obvious damage or inflammation in the major organs (i.e. Liver, spleens, kidneys, hearts, and lungs). The aggregation of Fe_3_O_4_-MNPs was mainly found in the livers and spleens, consistent with the normal distribution pattern of substances injected intravenously^41^. NAC administration did not change the distribution or the aggregation of Fe_3_O_4_-MNPs in the major organs (Fig. 5).

**Figure 6:**
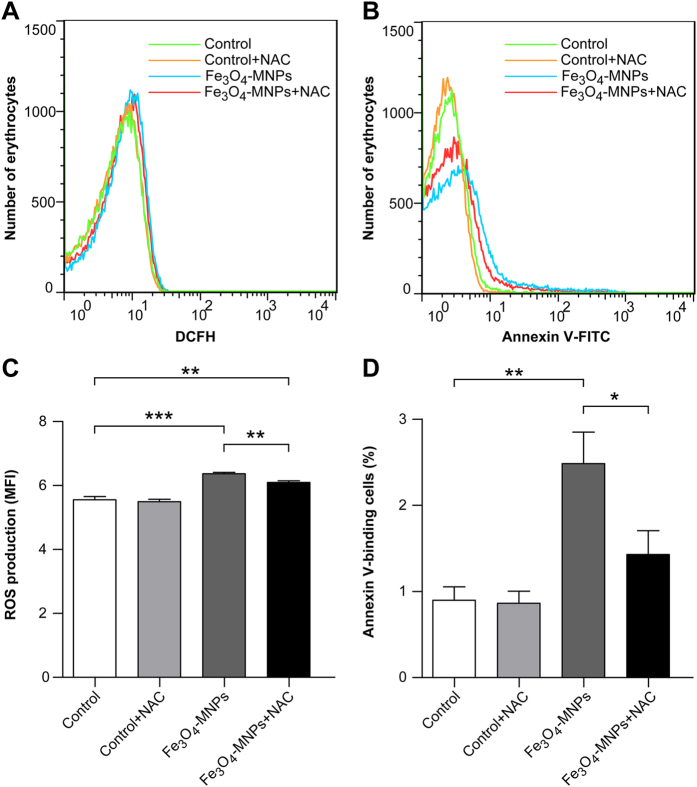
Cytosolic ROS and phosphatidylserine exposure of erythrocytes from rats. Cytosolic ROS (**A,C**) and phosphatidylserine exposure (**B,D**) of erythrocytes from rats injected with saline, saline with NAC, Fe_3_O_4_-MNPs, and Fe_3_O_4_-MNPs with NAC. Blood samples collected from tail vein were immediately stained by fluorescent Probe-DCFH-DA and Annexin-V-FLUOS and analyzed by flow cytometry. Values represent means ± SEM, n = 7, *p < 0.05, **p < 0.01, and ***p < 0.001.

Compared with the control groups, Fe_3_O_4_-MNP injection significantly increased ROS production, consistent with the *in vitro* results (Fig. 6A,C). The percentage of PS-displaying erythrocytes was less than 1% in the control groups. With Fe_3_O_4_-MNP injection, this percentage increased to 2.48%, meaning that erythrocyte degradation and regeneration was accelerated roughly 2-fold (Fig. 6B,D). NAC significantly inhibited Fe_3_O_4_-MNP-induced increases in ROS and phosphatidylserine exposure, although they were still higher than those of the control groups (Fig. 6A–D). These results suggested that Fe_3_O_4_-MNPs injection in rats induced eryptosis *in vivo* via ROS production and NAC could alleviate the oxidative stress to some extent.

The majority of the clinical chemistry parameters were within the normal ranges and showed no difference between treated- and the control groups, suggesting limited systemic toxicity caused by the doses investigated in this study (Figure S 6). Only the value of Total bilirubin (TBIL) in Fe_3_O_4_-MNP-treated group showed a significant increase compared with that of the control group (*p* < 0.05) and NAC administration inhibited Fe_3_O_4_-MNP-induced increase of TBIL, falling down within the normal range (Figure S 6). TBIL level is related to the degradation of senescent or injured erythrocytes, and fluctuations in this value suggest that Fe_3_O_4_-MNP injection can lead to excess erythrocyte degradation that can be alleviated by NAC.

During hematology analysis, the majority of hematology markers relevant to erythrocytes displayed significant difference between the control group and the nanoparticle injected group, such as RBC counts, hemoglobin, mean corpuscular volumes, mean corpuscular hemoglobin, and red cell distribution widths (Figure S 7). The values of all of these markers were within the normal range except hemoglobin (Figure S 7). Decreases of RBC counts, hemoglobin, and mean corpuscular hemoglobin indicated an excessive degradation of erythrocytes. There was also a decrease in mean corpuscular volumes and red cell distribution widths, which reflected a more uniform size and indicated that there were an enormous number of erythrocytes to be generated. However, NAC administration did not reverse changes of these erythrocyte-relevant markers. These results suggested that Fe_3_O_4_-MNP injection might lead to a mild to moderate risk for anemia.

**Figure 7:**
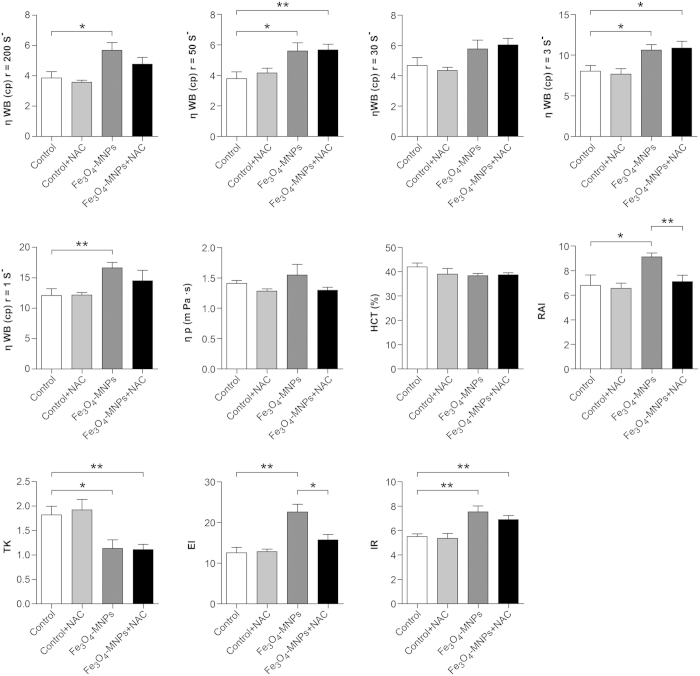
Results of hemorheology of rats injected with saline, saline with NAC, Fe_3_O_4_-MNPs, and Fe_3_O_4_-MNPs with NAC. Viscosity of whole blood (ηWB), viscosity of plasma (ηp), RBC aggregation index (RAI), erythrocyte deformation index (TK), erythrocyte electrophoresis time (EI), and erythrocyte rigidity index (IR). Values represent means ± SEM, n = 7, *p < 0.05, **p < 0.01.

The impact of nanoparticles on the flow properties of blood was evaluated by hemorheology. The hematocrit and plasma viscosities were not statistically significant. However, the indices related to the mechanical properties of erythrocytes differed significantly between the control and the Fe_3_O_4_-MNPs groups, such as erythrocyte electrophoresis time, RBC aggregation index, erythrocyte deformation index, erythrocyte rigidity index, and the viscosity of whole blood. The changes in RBC aggregation index and erythrocyte electrophoresis were inhibited by NAC administration (Fig. 7). These findings demonstrated that Fe_3_O_4_-MNPs treatment led to a decrease in deformability and changes in hemorheology, which would contribute to microcirculation disturbance and tissue damage by directly blocking capillaries and potential thrombogenesis^42^. Altogether, the results *in vivo* suggested that although there was a risk of anemia or thrombus formation, there was no obvious systemic toxicity risk after the administration of nanoparticles.

## Discussion

Developing an assessment system, in addition to hemolysis, with sensibility, predictability, and reliability is critical to detect the cytotoxicity of Fe_3_O_4_-MNPs^43^. Currently, evaluation of the hemocompatibility of nanoparticles mainly relies on hemolysis analysis, which is not delicate enough to reflect the full spectrum of erythrocyte injuries. However, determination of the hematologic toxicity of Fe_3_O_4_-MNPs on alternations of cellular membrane structure and function of erythrocytes remains unexplored. Thus, a predictive parameter beyond hemolysis testing is highly desirable. In this study, we developed a reliable system with eryptotic indices to evaluate the erythrocyte compatibility with Fe_3_O_4_-MNPs and to determine hazards (e.g. anemia and thrombogenesis) associated with the MNPs even at safe doses.

In the present study, our data demonstrated that eryptosis analysis was more sensitive in measuring hemocompatibility of nanoparticles for erythrocyte injuries, compared with currently used chemolysis for hemolysis. Currently, the safe and non-cytotoxic concentration is up to 100 μg/ml based on hemolysis standard^44^. Even when the concentration of nanoparticles rises to up to 400 μg/ml, the hemolysis rate of modified magnetic nanoparticles is less than 2%^43^. However, our data showed that Fe_3_O_4_-MNP caused phosphatidylserine externalization was statistically higher in the treated group than in the non-treated group when the dosage of nanoparticles is 25 μg/ml *in vitro*. Previous studies have shown that phosphatidylserine externalization evaluation is more sensitive than hemolysis testing^30^,^31^,^32^. Eryptosis is presumably a physiological protective mechanism to eliminate injured or defective erythrocytes to forestall hemolysis and the release of hemoglobin^14^,^45^. Phosphatidylserine acts as a key role in cell cycle signaling, specifically in relationship to apoptosis. When exposed to Fe_3_O_4_-MNP *in vitro*, the percentage of phosphatidylserine exposed on the cell surface reached over 3-fold greater than the hemolysis rate in this study. Then the phagocytosis and subsequent degradation of cells with exposed phosphatidylserine are inevitable.

**Figure 8:**
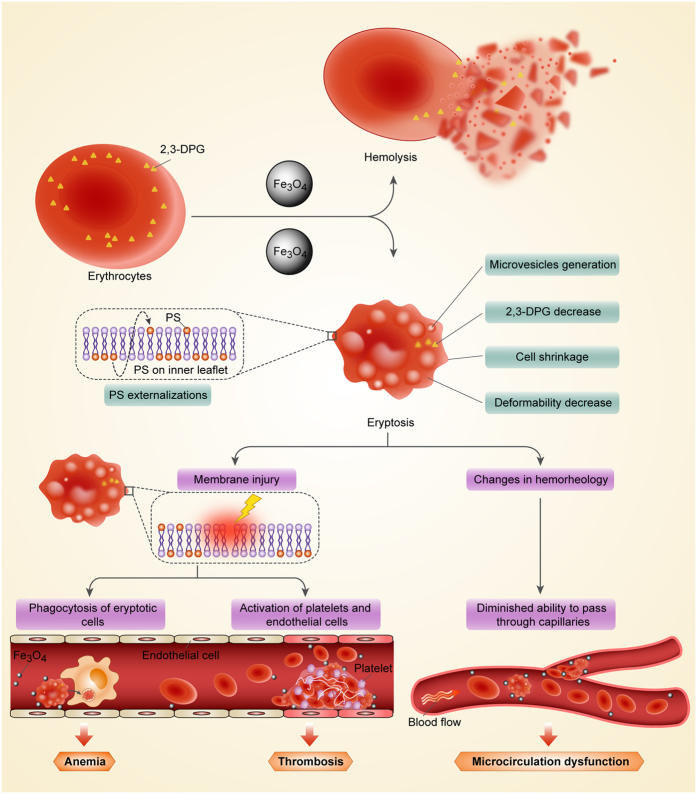
Schematic illustration of Fe_3_O_4_-MNPs impacts on erythrocytes and circulation.

*In vivo*, accelerated eryptosis leads to erythropenia, anemia and impairment of microcirculation. Normally, the loss of eryptotic erythrocytes is compensated by stimulation of erythropoiesis^37^. However, the results showed that Fe_3_O_4_-MNPs exposure caused excessive eryptosis could not be fully compensated by erythropoiesis. This is because numbers of erythrocytes and the hemoglobin concentration were significantly lower in the Fe_3_O_4_-MNP treated groups compared with the non-treated group. Particularly, for hemoglobin concentrations, Fe_3_O_4_-MNP administration resulted in a sharp decline beyond the normal ranges, suggesting a significant risk for anemia. In hemorheologcal analysis, markers relevant to the mechanical properties of erythrocytes decreased with Fe_3_O_4_-MNP treatment. These outcomes can lead to a risk of ischemia or hypoxia caused by microcirculation blood hypoperfusion (Fig. 8)^46^. Interestingly, N-acetylcysteine administration could not alleviate the acceleration of eryptosis and erythrocyte degradation, although it may be useful to ameliorate defects in some mechanical properties, such as whole blood viscosity (WBV), RBC aggregation index (RAI), and erythrocyte electrophoresis time (EI).

Furthermore, evaluations based on eryptosis can better predict the fate of the erythrocyte in circulation and can also reflect the risks posed by damaged erythrocytes that disrupt circulation. Phosphatidylserine is restricted to the cytoplasmic leaflet of the plasma membrane. However, it was shifted to the outer cellular membrane induced by Fe_3_O_4_-MNPs exposure, where it is recognized by specific phagocytic receptors, such as T-cell immunoglobulin mucin receptor 4 (TIM4). Therefore, cells with exposed PS were rapidly engulfed and degraded by macrophages^47^,^48^. Systematic administration of Fe_3_O_4_-MNPs induced 2% of erythrocytes’ phosphatidylserine externalization, which was lower that *in vitro*. It is attributed to one remarkable fact that the Annexin-V-FLUOS labeled erythrocytes represented only a fraction of eryptotic cells, since phosphatidylserine externalization is recognized and rapidly engulfed and degraded by macrophages. In fact, the percentage was over 2-fold greater and statistically higher than those of phosphatidylserine externalization in the control group. Membrane blebbing accounts for nearly 20% loss of membrane surface area, leading to a shape transformation and a reduction in deformability. Excessive blebbing accelerated the aging processes of erythrocytes^49^. ROS production resulted in the degradation of band 3 and spectrin and the opening of cation channels. It is critical for maintaining RBCs morphology, plasticity, and osmotic stability^50^. The increased cytosolic Ca^2+^ affected the skeleton flexibility and stability, intracellular ion balance, and facilitates phosphatidylserine externalization^51^. Membrane blebbing, phosphatidylserine externalization, shape transformation and reduction in deformability contributed to the phagocytic uptake and mechanical properties changes, which were reflected by hematology and hemorheology alternation after the treatment of Fe_3_O_4_-MNPs. Furthermore, microvesicles generated by blebbing and externalized phosphatidylserine have procoagulant activity suggesting a hazard of thrombin generation^15^,^52^. Therefore, the acceleration of eryptosis induced by Fe_3_O_4_-MNPs should not be overlooked and warrants further investigation. Overall, some hallmarks of eryptosis may represent more sensitive and predictive parameter of toxicity compared with hemolysis.

Recently, the interaction between nanoparticles and erythrocytes has been investigated^25^,^53^. However, the possible mechanism underlining the Fe_3_O_4_-MNP induced eryptosis was still not clear. There are reports that the increases of intracellular ROS might cause the potential toxicity in eryptosis^37^,^54^. Oxidative stress induces activation of Ca^2+^-permeable nonselective cation channels. With Ca^2+^ influx, erythrocytes suffered from PS externalization, cellular membrane blebbing, the loss of deformability, and clearance by macrophages. Our results confirmed that ROS production was the original and pivotal trigger of eryptosis. N-acetylcysteine, a ROS inhibitor, effectively blocked the eryptotic processes. Ca^2+^ influx is also a powerful trigger of eryptosis. Inhibition of Ca^2+^ entry relieved the phosphatidylserine externalization, consistent with previous studies^14^,^51^,^55^. However, the loss of membrane phosphatidylserine asymmetry was calcium-independent. The significant increase in cytosolic calcium concentrations occurred after ROS production, while Ca^2+^ influx was remarkably influenced by oxidative stress. Consequently, with ROS generation and Ca^2+^ entry promoted the eryptosis caused by Fe_3_O_4_-MNPs.

There is mounting evidence that the impacts of nanoparticle exposure on erythrocytes include a series of subtle changes in ionic balance, energy metabolism, physiology and rheological properties, in addition to hemolysis. As a result, there are often conflicting data between hemolysis testing *in vitro* and blood-compatibility *in vivo*. The injured erythrocytes progress to eryptosis, lose capability of oxygen delivering, impeding microcirculation, and are cleared from circulating blood at last. These changes, as correlated with some specific clinical disorders, can be detected by eryptotic indices *in vitro*. Consequently, eryptotic indices seem to be reasonable prediction metrics for predicting how nanoparticles impact erythrocytes and blood circulation *in vivo* (Fig. 8).

## Conclusions

In summary, we reported that 25 ug/ml Fe_3_O_4_-MNPs caused significant damage to erythrocytes in *in vitro* experiments and 12 mg/kg Fe_3_O_4_-MNPs lead to apoptosis of circulating erythrocytes *in vivo*. Erythrocyte injury of Fe_3_O_4_-MNPs can be divided into the early and later phages, eryptosis and hemolysis. Eryptosis does not induce acute hemolyzation characterized by hemoglobin releasing; it leads pathological alternations on cellular membrane and erythrocyte dysfunction. In this study, we demonstrated that Fe_3_O_4_-MNPs cause programmed cell death in erythrocytes with pathological changes on cellular membrane, abnormal cytosolic calcium levels, and oxidative stress and changes in the mechanical property of erythrocytes *in vitro* and *in vivo*. This study indicates that phosphatidylserine exposure, the index of eryptosis, can serve as a sensitive and reliable predictor for erythrocyte injury, while monitoring the nanotoxicity of the nanoparticles when systemically administrated. In addition, these metrics provide potential in determining the hazards of new types of nanoparticles or other biomaterials for clinical applications.

## Materials and Methods

### Materials

FeSO_4_·7H_2_O, KNO_3_, and KOH were purchased from Alfa Aesar Co. (China). Annexin-V-FLUOS was provided by Roche (Germany). Fluo-3/AM was supplied by Invitrogen (USA). An ATP assay kit and 2′,7′-dichlorofluorescin diacetate (DCFH-DA) were purchased from Beyotime (China). Human 2,3-DPG (2,3-Disphosphoglycerate) ELISA Kit was purchased from USCNLIFE (China). Phorbol 12-myristate 13-acetate (PMA), 1,1′-Dioctadecyl-3,3,3′,3′-tetramethylindocarbocyanine perchlorate (DiI), N-2-hydroxyethylpiperazine-N-2-ethane-sulfonic acid (HEPES), N-acetylcysteine (NAC) and 4,6-Diamidino-2-phenylindole dihydrochloride (DAPI) were obtained from Sigma-Aldrich (USA). Fetal bovine serum (FBS) was purchased from Gibco (AU). Penicillin-Streptomycin and L-Glutamine were provided by Gibco (USA). All Ringer solution and other salt solutions were freshly prepared before each experiment.

### Preparation and characterization of Fe_3_O_4_-MNPs

Fe_3_O_4_-MNPs were prepared according to a previously reported method with some modifications^56^. Briefly, 10 ml of 2 M KNO_3_ and 10 ml of 0.5 M KOH were added to flask and nitrogen was bubbled into the flask, then 10 ml of 0.1 M FeSO_4_ solution was added. The flask was immersed in an oil bath at 90 °C for 4 h. The precipitate (Fe_3_O_4_-MNPs) was washed by distilled water, freeze-dried and preserved in a drier. The morphology and diameter distribution were observed by transmission electron microscopy (TEM) using a JEm-1400 (JEOL, Japan) and dynamic light scattering (DLS) using a NanoZS90 Zetasizer (Malvern Instruments Co. Ltd., UK). The UV-vis absorption spectra of Fe_3_O_4_-MNPs was evaluated using a TU-1901 UV spectrophotometer (BEIJING PUXI General Instruments, China) with a slit dimension of 2.0 nm and a quartz cuvette with inner size at 10 mm x 10 mm and outside size at 12.5 mm x 12.5 mm.

### Erythrocyte preparation

This study was approved by the Medical Ethics Committee of The Second Hospital affiliated with The Third Military Medical University. All procedures were conducted in accordance with the approved guidelines of the Medical Ethics Committee of The Second Hospital affiliated with The Third Military Medical University. All human subjects involved gave written informed consent. Leukocyte-free erythrocytes from healthy donors were used shortly after collection and were provided by the Chongqing Blood Centre. Hematocrit was adjusted to 0.4% with Ringer solution (125 mM NaCl, 32 mM HEPES, 5 mM glucose, 5 mM KCl, 1 mM MgSO4, 1 mM CaCl_2_, pH7.4), and all incubation condition of erythrocytes were 37 °C, 5% CO_2_, with 95% humidity. Calcium-free Ringer solution was made by using 1 mM EGTA as a substitute for 1mM CaCl_2_.

### Hemolysis measurement and blood gas analysis

To determine the impact of Fe_3_O_4_-MNPs on hemolysis, RBCs were incubated with Fe_3_O_4_-MNPs at the concentrations from 3.125 to 1600 μg/ml for 3 h and 24 h. Erythrocytes incubated with deionized water and Ringer solution were used as positive (+) and negative (−) controls, respectively. After centrifuged at 10,016 × g for 3 min, supernatants were collected for spectrophotometry. The absorbance (*A*) was analyzed at 570 nm with a reference at 655 nm in a Varioskan Flash Multimode Reader (Thermo Fisher, USA). The percentage of hemolysis was calculated as: 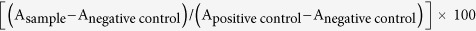
57. The UV-vis absorption spectra of supernatant were measured with a slit dimension of 5.0 nm. Erythrocytes were incubated with 200 μg/ml Fe_3_O_4_-MNPs for 48 h. Blood gas detecting indices were analyzed using a Critical Care Xpress Blood Gas Analyzer (Nova, US).

### Measurement of phosphatidylserine exposure

To evaluate the effects of Fe_3_O_4_-MNPs on eryptosis, the externalization of phosphatidylserine was detected by fluorescent Annexin V conjugates. After washed with Ringer solution, erythrocytes were resuspended in 250 μl incubation buffer with 5 μl Annexin-V-FLUOS at 20 °C for 15 min. The forward scatter (FSC) and Annexin-V-FLUOS fluorescence intensities were measured by FACS Calibur (BD, USA). The percentage of phosphatidylserine unmasking in erythrocytes was analyzed using Flowjo (Treestar, USA). Images were obtained using confocal microscopy (Carl Zeiss, 510 meta, Germany).

### Scanning electron microscopy (SEM)

To characterize the interaction between Fe_3_O_4_-MNPs and erythrocytes, SEM was employed to observe the transformation, blubbing, and pores of cellular membrane. Erythrocytes were incubated with Fe_3_O_4_-MNPs (200 μg/ml) for 12, 24, and 48 h. Erythrocytes treated with Ringer solution for 12 h were used as a control. The cells were gold-coated with a JEOL JEC-3000FC and observed using a Hitachi S-3400N.

### Cell culture and cell uptake assay

THP-1 cells were obtained from the American Type Culture Collection. 2 × 10^6^ cells were pre-activated by PMA 10 ng/ml for 24 h. Differentiated THP-1 cells were washed by complete RPMI-1640 medium and cultured for another 48 h before phagocytosis^58^. Erythrocytes were incubated with Fe_3_O_4_-MNPs (200 μg/ml) or with Ringer solution (negative control) for 12 h, then labeled by DiI and washed by PBS. Differentiated THP-1 cells were incubated with labeled erythrocytes for 120 min. After incubation, THP-1 cells were washed and counterstained with DAPI^59^. Finally, the images were obtained by CLSM z-stack scanning and analyzed using 3D reconstruction.

### Measurement of intracellular ROS, Ca^2+^, 2,3-DPG, ATP, and RBC deformability

Intracellular ROS*, Ca*^*2*+^ were monitored using fluorescent probes DCFH-DA and Fluo-3 AM, respectively. The changes in fluorescence were measured using FACS Calibur in fluorescence channel FL-1. The geometric means of fluorescence intensity were analyzed by Flowjo software. Intracellular 2,3-DPG and ATP concentrations were detected using Human 2,3-DPG ELISA Kit and ATP assay kit, respectively. The RBC deformability was monitored using an erythrocyte deformability analyzer (LBY-BX, China).

### Toxicity of Fe_3_O_4_-MNPs and protective effects of N-acetylcysteine *in vivo*

The toxicity of Fe_3_O_4_-MNPs and the protective effects of N-acetylcysteine were investigated in a rodent model. The animal received concentration of 12 mg/kg/injection of Fe, a dosage generally considered to be safe^12^. All procedures were performed in accordance with protocols approved by the Animal Management Rules of the Ministry of Health of the People’s Republic of China (Document NO. 55, 2001). Female CD^®^ IGS Rats, aged 8 weeks, weighing 190 ± 10 g were obtained from Beijing Vital River Laboratories (China). Twenty-eight rats were randomly assigned to the following four groups (G) (n = 7/G): **G1**, the control group in which animals were injected with 1 ml saline; **G2**, the control + N-acetylcysteine group in which animals were injected with 1 ml saline with N-acetylcysteine (2 g/L) administered in distilled water; **G3**, the Fe_3_O_4_-MNPs group in which Fe_3_O_4_-MNPs were injected at a dosage of 12 mg/kg of Fe by intravenous injection; **G4,** the Fe_3_O_4_-MNPs + N-acetylcysteine group in which animals received Fe_3_O_4_-MNPs at a dosage of 12 mg/kg of Fe by intravenous injection and were administered N-acetylcysteine (2 g/L) in distilled water. Rats were intravenously treated with saline or Fe_3_O_4_-MNPs every other day for a total of three treatments. N-acetylcysteine was administered during the experimental period.

At day 6, animals were anaesthetized using isoflurane. Blood samples were collected and used to measure ROS, phosphatidylserine exposure, hematology analysis, blood serum biochemistry and hemorheology analysis. The normal ranges of biochemistry and hematology data of healthy female CD^®^ IGS Rats were obtained from Charles River Laboratories (http://www.criver.com/files/pdfs/rms/cd/rm_rm_r_cd_rat_clinical_pathology_data.aspx). The major organs were stained with hematoxylin and eosin (H&E). The distribution of Fe_3_O_4_-MNPs and the morphology of organs were observed under a light microscope (Olympus BX63, Japan).

In order to investigate pharmacokinetic characteristics of Fe_3_O_4_-MNPs, we measured blood Fe ions concentration after Fe_3_O_4_-MNPs injection using ICP-OES. Eight Rats were randomly assigned to two groups (n = 4/G): **G’1**, the control group in which animals were injected with 1 ml saline; **G’2**, the Fe_3_O_4_-MNPs group in which Fe_3_O_4_-MNPs were injected intravenously at a dosage of 12 mg/kg of Fe. Blood samples were obtained from G’1 group and G’2 group at 5, 10, 20, 30, 60, 120, 180, 240, 360, 480, 720, and 1440 min after the last injection. Blood Fe ions concentration was measured using iCAP 6000 SERIES (Thermo SCIENTIFIC, USA) after acid hydrolysis.

### Statistical analysis

All data were expressed as mean ± SEM (standard error of the mean). The results were analyzed using GraphPad Prism 5 software (GraphPad Software Inc., CA). A *p*-value < 0.05 was considered statistically significant.

## Additional Information

**How to cite this article**: Ran, Q. *et al.* Eryptosis Indices as a Novel Predictive Parameter for Biocompatibility of Fe_3_O_4_ Magnetic Nanoparticles on Erythrocytes. *Sci. Rep.* **5**, 16209; doi: 10.1038/srep16209 (2015).

## Supplementary Material

Supplementary Information
