# Supplementary material for: Eryptosis Indices as a Novel Predictive Parameter for Biocompatibility of Fe3O4 Magnetic Nanoparticles on Erythrocytes
Source: Sci Rep. 2015 Nov 5;5:16209. doi: 10.1038/srep16209 (PMC4633654; doi:10.1038/srep16209)
Supplement: Supplementary Information [file srep16209-s1.doc]

**Supplementary Data**

**Eryptosis Indices as a Novel Predictive Parameter for Biocompatibility of Fe_3_O_4_ Magnetic Nanoparticles on Erythrocytes**

Qian Ran^1^, Yang Xiang^1^, Yao Liu^2^, Lixin Xiang^1^, Fengjie Li^1^, Xiaojun Deng^1^, Yanni Xiao^1^, Li Chen^1^, Lili Chen^1^, and Zhongjun Li^1^*

^1^Department of Blood Transfusion, The Second Affiliated Hospital, Third Military Medical University, Chongqing, China

^2^Department of Hematology, The Second Affiliated Hospital, Third Military Medical University, Chongqing, China.

*Correspondence: Zhongjun Li

Department of Blood Transfusion, The Second Affiliated Hospital, Third Military Medical University, Xinqiao Road, Shapingba, Chongqing 400037, China

E-mail: [johnneyusc@gmail.com](http://cn.mc158.mail.yahoo.com/mc/compose?to=johnneyusc@gmail.com)

Tel: +86-23-68755319; Fax: +86-23-68755319


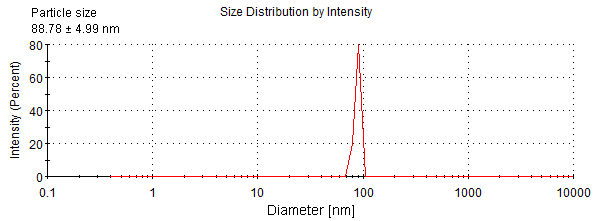


**Figure S 1.** Measurement of Fe_3_O_4_-MNPs size by dynamic light scattering


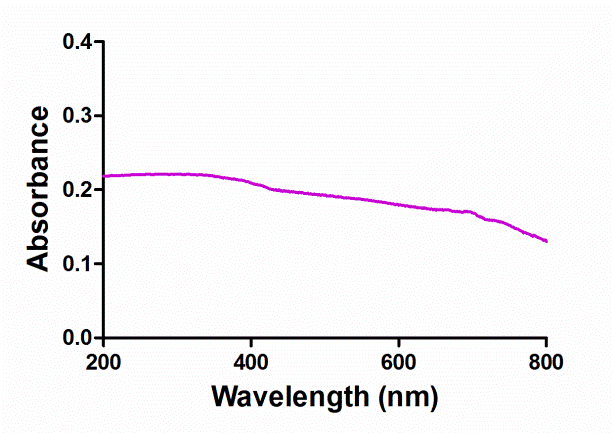


**Figure S 2.** UV-vis absorption spectra of Fe_3_O_4_-MNPs


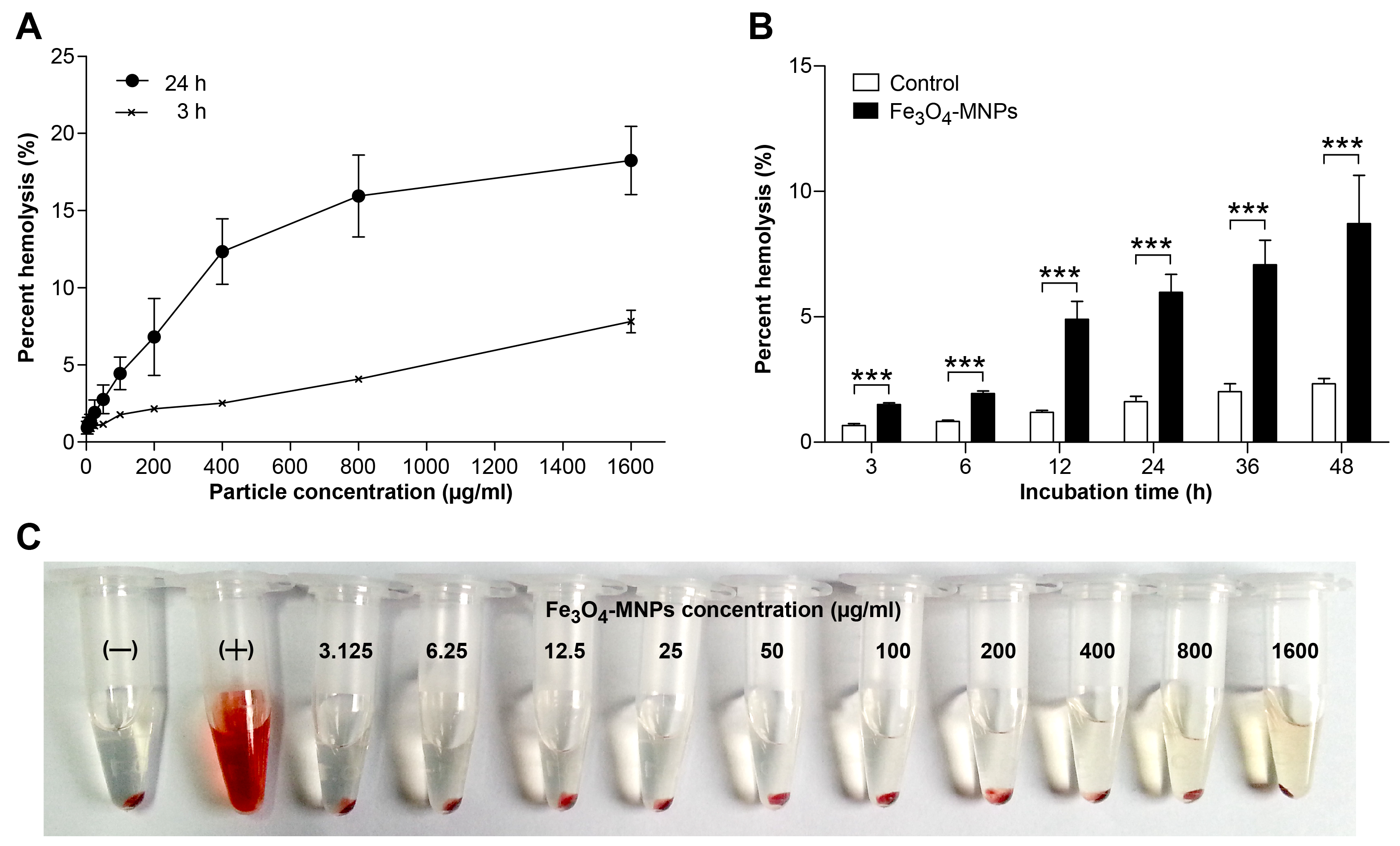


**Figure S 3. Hemolytic activity of Fe_3_O_4_-MNPs.** (A) The percentage of hemolysis of erythrocytes incubated with Fe_3_O_4_-MNPs at a series of concentrations ranging from 3.125 to 1600 μg/ml for 3 h and 24 h. (B) The percentage of hemolysis of erythrocytes incubated with 200 μg/ml Fe_3_O_4_-MNPs for different time points. (C) A photograph of hemolysis of erythrocytes incubated with 200 μg/ml Fe_3_O_4_-MNPs for 24 h. Values represent means ± SEM, n = 9, ****p* < 0.001.


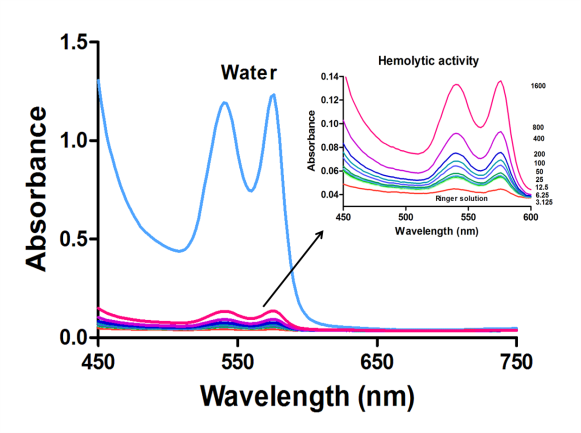

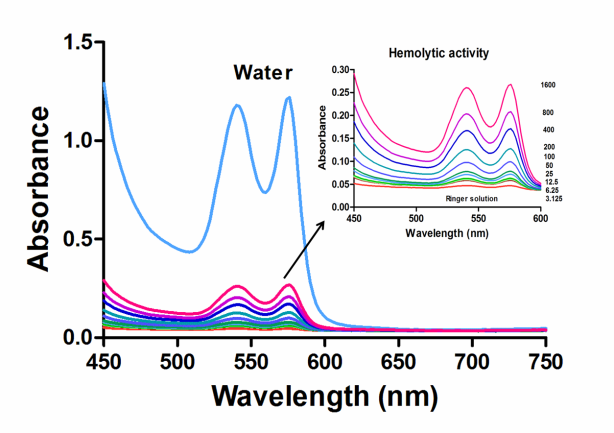


A B

**Figure S 4.** Hemolytic activity of Fe_3_O_4_-MNPs at 3 h (A) and 24 h (B) at different concentrations (3.125, 6.25, 12.5, 25, 50, 100, 200, 400, 800, 1600 μg/ml, respectively) using deionized water as a positive control and Ringer solution as a negative control. The upper-right insets show the enlarged UV-vis absorption spectra in each panel.


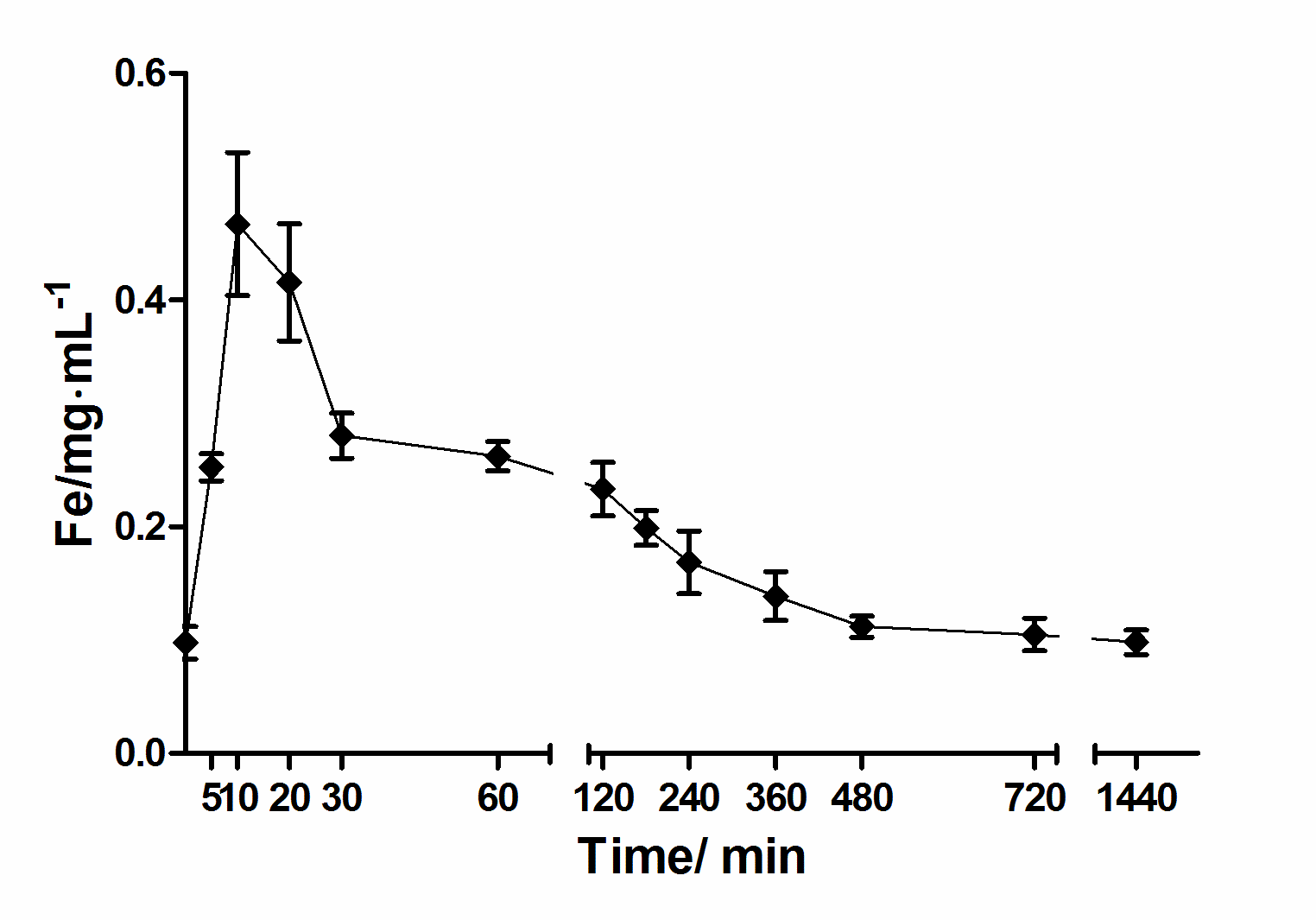


**Figure S 5.** Quantification of iron concentration in blood after Fe_3_O_4_-MNPs injection (5, 10, 20, 30, 60, 120, 180, 240, 360, 480, 720, 1440 min) by ICP-OES. Data are shown as mean ± SD, n = 4.





**Figure S 6. Results of blood serum biochemistry of rats injected with saline, saline with NAC, Fe_3_O_4_-MNPs, and Fe_3_O_4_-MNPs with NAC.** Urea (UREA), creatinine (CRE), Alanine aminotransferase (ALT), Aspartate aminotransferase (AST), Total protein (TP), Albumin (ALB), Globulin (GLB), Albumin/ Globulin (ALB/GLB), Total bilirubin (TBIL), Direct bilirubin (DBIL), Alkaline phosphatase (ALP), Lactic acid dehydrogenase (LDH), Cholesterol (CHOL), Blood glucose (GLU). Values represent means ± SEM, n = 7, **p < 0.05*.





**Figure S 7. Results of hematology of rats injected with saline, saline with NAC, Fe_3_O_4_-MNPs, and Fe_3_O_4_-MNPs with NAC.** White blood cells (WBC), Red blood cells (RBCs), Hemoglobin (HGB), Hematocrit (HCT), Mean corpuscular volume (MCV), Mean corpuscular hemoglobin (MCH), Mean corpuscular hemoglobin concentration (MCHC), Red cell distribution width CV (RDW-CV), Red cell distribution width SD (RDW-SD), Platelet (PLT), Mean platelet volume (MPV). Values represent means ± SEM, n = 7, **p < 0.05**p < 0.01, and ***p < 0.001*.

| **Table. S1. Arterial blood gas analysis of erythrocytes after 48-hour incubation with 200 μg/ml Fe_3_O_4_-MNPs** | | | |  | |
| --- | --- | --- | --- | --- | --- |
|  | **Control**  *(n=9 cases)* | **Fe_3_O_4_-MNPs**  *(n=9 cases)* | ***p* Value*** | | |
| **pH** | 7.0± 0.03 | 7.1 ± 0.02 | 0.064 | | |
| **pO_2_** (*mmHg*) | 168.4 ± 1.17 | 169.8 ± 1.21 | 0.481 | | |
| **Standard bicarbonate** (*mmol/L*) | 7.3 ± 0.03 | 9.5 ± 0.37 | 0.004****** | | |
| **SO_2_%** | 98.3 ± 0.13 | 98.9 ± 0.07 | 0.027***** | | |
| **K^+^** (*mmol/L*) | 5.4 ± 0.04 | 5.1 ± 0.08 | 0.047***** | | |
| **Ca^2+^** (*mmol/L*) | 1.0 ± 0.01 | 1.1 ± 0.01 | 0.921 | | |
| **Glucose** (*mmol/L*) | 3.5 ± 0.05 | 3.7 ± 0.03 | 0.025***** | | |
| **Urea** (*mmol/L*) | 1.4 ± 0.03 | 1.4 ± 0.02 | 0.465 | | |
| **PCO_2_** (*mmHg*) | 21.7 ± 0.14 | 18.9 ± 0.77 | 0.025***** | | |
| **TCO_2_** (*mmol/L*) | 5.9 ± 0.27 | 6.9 ± 0.38 | 0.118 | | |
| **HCO_3_^-^** *(mmol/L*) | 5.3 ± 0.28 | 6.5 ± 0.18 | 0.017***** | | |
| **Na^+^** (*mmol/L*) | 133.5 ± 0.71 | 135.1 ± 0.98 | 0.199 | | |
| **Cl^-^** (*mmol/L*) | 124.1 ± 0.36 | 121.8 ± 0.33 | 0.004** | | |
| **Mg^2+^** (*mmol/L*) | 0.8 ± 0.02 | 0.8 ± 0.02 | 0.115 | | |
| ***Note****: Values represent means ± SEM;* ******* *p<0.05; **p<0.01* | | | | |  |
